# Stakeholders’ perspectives on a digital myopia screening program in children: a qualitative analysis

**DOI:** 10.3389/fopht.2025.1585320

**Published:** 2025-07-08

**Authors:** Casper van der Zee, Janneau L. J. Claessens, Petra T. Rausch-Koster, Saskia M. Imhof, Ruth M. A. van Nispen, Robert P. L. Wisse, Hilde P. A. van der Aa

**Affiliations:** ^1^ Ophthalmology Department, University Medical Center Utrecht, Utrecht, Netherlands; ^2^ Department of Ophthalmology, Bergman Clinics, Naarden, Netherlands; ^3^ Amsterdam University Medical Center (UMC), Vrije Universiteit Amsterdam, Ophthalmology, Amsterdam, Netherlands; ^4^ Amsterdam Public Health Research Institute, Amsterdam, Netherlands; ^5^ Easee BV, Amsterdam, Netherlands

**Keywords:** stakeholder analysis, online myopia screening, children, Dutch health care system, refractive screening, telemedicine, qualitative study, health policy

## Abstract

**Purpose:**

This study was aimed at identifying barriers and opportunities to use a self-administered online refractive eye test by various stakeholders of a pediatric vision screening program.

**Methods:**

This qualitative study performed semi-structured interviews with myopic children and their parents, eye care professionals, and policymakers. Three topic lists were developed, delineating themes to identify gaps, barriers, and opportunities. Interviews were anonymously recorded, transcribed, and coded using thematic analysis. Quantitative data was acquired from a concomitant clinical validation study.

**Results:**

In total, 14 interviews were conducted, of which seven were with children and their parents, four with eye care professionals, and three with policymakers. The patients and parents were positive about the instructions and age appropriateness. They noted that the test could be designed as more child-friendly and preferred receiving feedback during the test. Eye care professionals and policymakers saw potential for using the test in children aged ≥12 without high refractive errors, yet they also underlined the false-positives rates, impacting care demand and costs. The population refraining from participation was expected to have higher health gains, yet including them was expected to be challenging without facilitating awareness.

**Conclusions:**

This qualitative study shows the perspectives for an online pediatric refractive screening. The patients and parents were open to self-administered screening and suggested improvements. The eye care professionals and policymakers were receptive to screening but also cautious, highlighting costs and scientific reliability. For better implementation, the policymakers underlined the relevance of the screening criteria, while the eye care professionals recommended targeting a specific population at risk that benefits most rather than screening the whole population.

## Introduction

Recently, a web-based test for self-assessing visual acuity and refractive errors in myopic children was evaluated for its reliability (1). The study demonstrated that children could successfully complete the test with parental supervision and highlighted areas for future improvement in accuracy. However, to understand the broader societal value of such a tool, it is crucial to explore the barriers and opportunities identified by stakeholders directly involved (2). To address this, we conducted a follow-up study to gather insights from stakeholders in the context of an online screening program.

Screening for myopia in children could be relevant as it increases awareness and potentially facilitates early detection (3). Thereby, myopic progression could be delayed, retaining as much sight and reducing as much complications as possible (4–6). Traditionally, this is achieved through physical screening programs in schools or community settings. However, these programs are demanding on resources and healthcare personnel. A digital remote self-assessment could serve as an auxiliary scalable and scalable tool.

This study explores the perspectives of patients (children and their parents), eye care professionals, and policymakers to use a digital test as a screening tool to detect myopia in a pediatric population aged 6 years or older. In this context, policymakers are defined as public decision-makers at governmental or organizational levels who are responsible for designing, implementing, and overseeing health programs, including those related to vision screening. Their role is essential in establishing guidelines, securing funding, integrating screening tools into national or regional health strategies, and promoting equitable access to care. By understanding their perspectives—alongside those of clinicians and end-users—this study aims to identify barriers and opportunities for the adoption of a remote screening program for refractive errors using an online eye test in The Netherlands. Thereby, it assesses stakeholders’ perspectives on how a digital screening could contribute to support early detection and increased awareness of myopia.

## Methods

**Figure 1:**
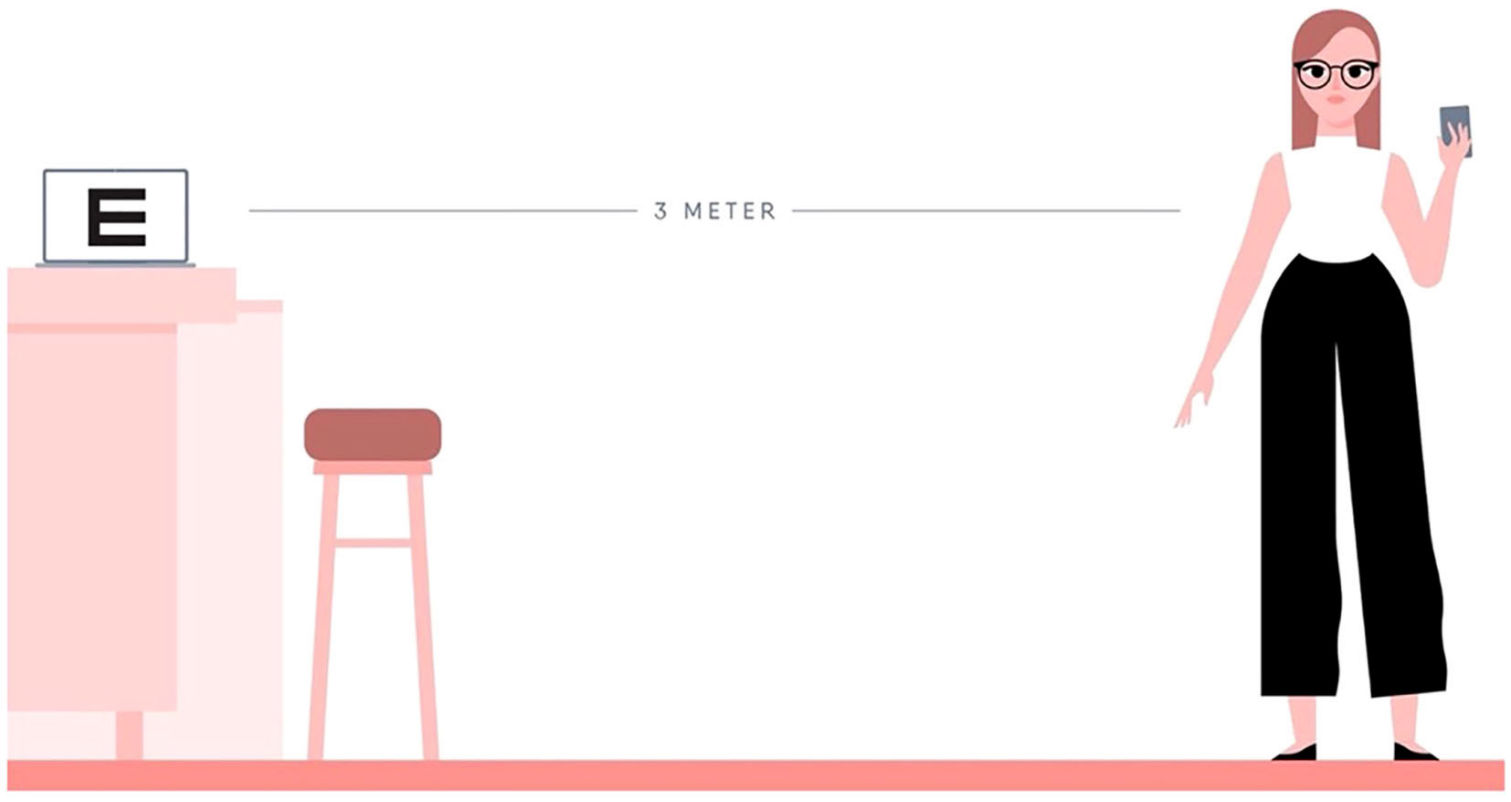
Online test set-up. A computer, distance of 3 m, and a phone are required to conduct the test.

This qualitative interview study originated from a clinical method comparison study evaluating an independent digital visual acuity and refractive error assessment in myopic children. The Medical Ethics Review Committee Erasmus MC approved this study (MEC-2021-0816). This study was conducted in accordance with the Declaration of Helsinki. Written informed consent was obtained from all participating children (called patients in this paper) and from their parent(s) or caregiver(s). The method of the comparison study was previously described in depth (1). Besides the methodology, we described the validation and limitations of using this application as a way to measure refractive error. In summary, in the comparison study, we invited children ≥6 years to perform web-based eye tests twice at home, assisted by a parent. Data collection occurred between September 1, 2022 and June 30, 2023. The participants were invited for the second test 5 days after their first home test. To perform the test, the participants were in possession of a smartphone and tablet or computer (**Figure 1**). The patients were recruited from the myopia control clinic at the Erasmus Medical Centre and Radboud Medical Centre; both university clinics are located in The Netherlands. This study gave rise to qualitative research questions on whether the test could be used as a screening program. Thus, patients and their parents, eye care professionals, and policymakers were invited to expand on their perspectives in the current study. Interviews were performed between August 1 and November 1, 2023.

### Online eye test

The online eye test was developed by Easee BV, Amsterdam, The Netherlands. The self-administered test measured the visual acuity and refractive error at home by using a phone and a laptop. In minors, the test was supervised by a parent or caregiver. The preparation of the test included several steps (**Figure 1**). First, the computer screen was calibrated so that optotypes were shown in the correct size. Then, the laptop was positioned at a 3-m distance and connected to a mobile phone. The patients were instructed to use a measuring tape or, alternatively, they could provide their shoe size and the number of heel-to-toe steps from the screen until a distance of 3 m was measured by the test. The patients were instructed to place a chair at that spot to ensure that they remained at the correct distance. Afterward, the optotypes and astigmatism dials were displayed on the screen. The phone functions as a remote control. Visual, written, and audio instructions were given before, during, and after the test. As a reference, the test time including set-up phase was previously assessed in an elderly population to be, on average, 18 min (SD ± 4) for both eyes (up to 93 years old; mean, 71 years) (7).

### Data collection

The identified stakeholder groups were divided into two groups (1): patients and parents and (2) eye care professionals and policymakers. All participants from the concomitant study who performed the test within the last 3 months were invited via email for an interview. Similar to the visual acuity assessment at the clinic, the online test was performed while wearing their current existing glasses or contact lenses, effectively assessing the habitual or presenting acuity. For clarity, visual acuity thus refers to presenting distance visual acuity (PDVA). Data on the demographics (**Table 1**) of all interviewees were collected. For patients, these were age, sex, school (primary, secondary), the spherical equivalent (diopters), the presenting distance visual acuity (LogMAR), current eyewear, use of atropine medication, and axial length of the eyes. For eye care professionals and delegates of policymakers, these were with regard to their profession and relevant background. The interviewees were interviewed in Dutch, and the interview took place within 6 months after performing the online test.

**Table 1:** Demographics of interviewees.

Interviewees	Variables
Patients (n=7)	
Age in years, mean (range)	12.3 (8–15)
Sex, n (%)	
Male	2 (29)
Female	5 (71)
School, n (%)^1^	
Primary	2 (29)
Secondary	5 (71)
SEQ in Diopters, mean (SD)^2^	
Right eye	-4.48 (1.57)
Left eye	-4.79 (2.00)
PDVA^3^ in LogMAR, mean (SD)	
Right eye	0.00 (0.04)
Left eye	0.01 (0.05)
Current eyewear, n (%)	
Glasses	3 (43)
Contact lenses	4 (57)
Atropine use, n (%)	
High-dose atropine (≥0.5%)^4^	3 (43)
Low-dose atropine (<0.5%)^4^	2 (29)
No atropine	2 (29)
Axial length in mm, mean (SD)	
Right eye	25.48 (0.68)
Left eye	25.45 (0.70)
Eye care professionals (n=4)	Ophthalmologist, academic (n=2)
	Optometrist, academic (n=1)
	Youth physician (n=1)
Delegates of Policymakers (n=3)	Public Health Organization ^a^ (n=1)
	Ministry of Health, Welfare and Sport ^b^ (n=1)
	Youth Healthcare Organization ^c^ (n=1)

All interviewees were from the Netherlands, eye care professionals and policymakers were affiliated with Dutch organizations. SEQ = Spherical Equivalent.

^1^Children usually start secondary school at the age of 12.

^2^Based on current spectacle prescription at baseline.

^3^Similar to the visual acuity assessment at the clinic, the online test was performed while wearing their current existing glasses or contact lenses, effectively assessing the habitual or presenting acuity. For clarity, visual acuity thus refers to presenting distance visual acuity (PDVA).

^4^One drop administered daily at night time in both eyes.

a GGD; Gemeentelijke Gezondheidsdienst.

b RIVM; Rijksinstituut voor Volksgezondheid en Milieu.

c TNO; T*oegepast Natuurwetenschappelijk Onderzoek*. Independent not-for-profit research organization.

The eye care professionals and policymakers were selected based on their status as key opinion leaders in the field or their affiliation with relevant organizations, as identified by the research team or other key opinion leaders who were interviewed. Three topic lists were developed, delineating themes and corresponding questions designed to identify gaps, barriers, and opportunities from their respective perspectives. The included topics were general perspectives, user experiences and social influences, trust in telemedicine and screening, clinical perspectives, and pitfalls, challenges, and effects of the screening program. This was subsequently supervised by experts in qualitative research from Amsterdam UMC (RN, HA, PR). Data was collected through semi-structured interviews to allow leeway for the interviewee to accentuate and elaborate on topics that they found important. This also gave the interviewees the possibility to introduce their own topics, if they were so inclined. All interviews were conducted via secured video calls (CZ and JC, both medical doctors) and direct supervision was provided (PR, a PhD–epidemiologist, with experience in qualitative research). All have experience in the field of ophthalmology. At the beginning of the interviews, the interviewees were provided with a presentation as a reminder of how the online eye test and the screening program worked. Quotes exemplifying relevant viewpoints of the interviewees were reported. These quotes were carefully translated into English by JC and CZ and subsequently supervised to ensure translational accuracy.

### Data analysis

Interviews were anonymously recorded, edited transcription was applied, and transcripts were imported into NVivo (release 14.23.0). The transcripts were analyzed thematically per stakeholder group (CZ and JC). A codebook was developed in advance of transcription based on the topic lists. Coding insights that emerged during transcription were added to the code book. The first transcript from each stakeholder was coded by both researchers (CZ and JC) to identify coding discrepancies, which were resolved through consultation with a third researcher if applicable (RW—for the codebook, see **Supplementary Table S1**). Descriptive statistics were analyzed using Microsoft Excel Version 16.89.1. The Consolidated Criteria for Reporting Qualitative Research (COREQ) was used to ensure a comprehensive and transparent description of the processes and details involved in both data collection and data analysis (8).

## Results

A total of 14 interviews were conducted, including myopic children and their parents (*n* = 7 children with nine parents), eye care professionals (*n* = 4), and policymakers (*n* = 3). The children were all Dutch and, on average, 12 years old (see **Table 1** for the descriptions). The identified themes were divided by (1) perspectives of patients and parents and (2) perspectives of eye care professionals and policymakers.

### Perspectives of patients and parents

#### Theme 1.1: Usability of the online test

The users thought that the instructions were clear. Most thought that the test was age appropriate, yet some noted that the test could be more child-friendly (**Table 2**, quote 1.1). All children finished the test with adequate motivation according to the parents, and all parents mentioned that supervision by them was necessary. The children needed help in setting up the test, e.g., finding the website link and measuring the 3-m distance. A possible improvement mentioned by one parent was that feedback could be given on how well the participants adhere to the test instructions, e.g., distance and staying motivated (quotes 1.2 and 1.3). None had problems with the fact that an eye care professional was not physically present during the test.

**Table 2:** Quotes exemplifying the viewpoints of the interviewees.

Theme	Quote (Q)^1^
Patients & Parents	
Theme 1: Usability of the online test	Q1.1: This might sound weird, but a little more decoration would help. That helps making the test more fun and keep up motivation. *11-year-old-male* c*hild*. Q1.2: I had the feeling that the test is not “complete”. That you have no feedback during the test, that makes it more difficult. In the clinic there is feedback, e.g. “You’re doing good, can you guess the next letter?” – *Mother of a 14-year-old-daughter*. Q1.3: People still have to follow the instructions well, you don't get any feedback on that, like standing at the right distance and not secretly looking through the fingers. Adults understand that too I think, but with children you have to supervise that, otherwise it is not reliable – *Mother of a 11-year-old son*.
Theme 2: General impression of the test as a screening method	Q1.4: We live 100 km away from the academic hospital. For an incidental or short check-up, this could be an interim solution in combined with a physical check-up every other year. That you have an interim check-up. And if something strange comes out of that, that you then get an email for a physical consultation – *Father of a 14-year-old daughter.* Q1.5: We also have an older daughter with myopia. Unfortunately, we found out about that too late, ultimately through school. Then you think, I would have liked to have known that much earlier – *Father of a 12-year-old daughter*.
Theme 3: Parties to distribute this screening method	Q1.6 We are quickly inclined to say: Why don't we do this at school? But I also realize that we are already throwing a lot on the school's plate. They see you coming…’ – *Father of a 14-year-old son.*
Theme 4: The uptake of screening by target group	Q1.7 So much is already asked of parents. You have to have a good story, create awareness. If you can clearly indicate what it means for a child later in life, in that case I think parents are more likely to be triggered to take action – *Mother of a 14-year-old daughter.* Q1.8: I think there is a group of skeptics against government agencies - and therefore also screening - that is growing. I think that group is perhaps the most difficult to catch – *Father of a 14-year-old son.*
Eye care professionals & Policymakers	
Theme 1: A case for digital screening	Q2.1: Something must happen, we can't have all these care resources manually performed by professionals. I think there are opportunities for technology to do something about this – *Eye Care Professional.*
Theme 2: Use case and screening update	Q2.2: I highly value the skills of orthoptists in vision measurements, especially in children younger than 12 years old. They guide children very well. I do not have the illusion that I can do that in a similar way – *Eye Care Professional.* Q2.3: It's not that who has over -2 Diopters remain unnoticed, I don't believe that in the Netherlands. So that one will seek care anyway. And after correction, patients most often join myopia control after that. – *Eye Care Professional*. Q2.4: I can imagine that even if the screening was free, parents would be reluctant since the consequence could be that they would have to buy expensive glasses afterwards, which could also deter people from the screening program. Momentarily, there is a personal contribution of 119 euros before the insurance steps in. – *Delegate of the Youth Healthcare Organization*. Q2.5: Of course, I prefer a child is earlier detected, e.g. at -0.5 Diopters instead of -1.5D, because they have lost one Diopter more of myopic progression we potentially could have prevented. But I also think that screening is difficult to get cost-effective. – *Eye Care Professional.*
Theme 3: Methodological design	Q2.6: We worry that false-positives will lead to unnecessary referrals and burden on the healthcare system, reducing capacity for patients who have eye complaints – *Eye Care Professional.* Q2.7 If the optician is not consulted after a positive online eye measurement, I expect that false-positives are going to burden the healthcare system unnecessarily – *Eye Care Professional.*
Theme 4: Practical barriers	Q 2.8: The WHO has fantastic rules for screening. Myopia could fit: the personnel is there for research, we have a good treatment, we prevent unnecessary vision loss. So I do believe that in the Netherlands we can cover these criteria, except for the last part: cost-effectiveness – *Eye Care Professional.* Q 2.9: For example, in Belgium they started screening with a *Plusoptix* device. The number of glasses has increased by 1/3. These are costs for the patients, but they are included in the cost-effectiveness analysis. So that suddenly makes screening more expensive as a society. You can't see that separately. I think that can be quite a pitfall. – *Eye Care Professional.*
Theme 5: Awareness	Q2.10: I think that as a healthcare professional you have an obligation to underline the necessity of lifestyle changes such as the 20-20-2 rule, which could have an effect on its own. On the other hand, my patients also have the desire to independently manage their own care, e.g. this eye measurement. In that case, you would rather offer something validated – *Eye Care Professional.* Q2.11: I think that remote screening already profits if it can raise public awareness his – *Delegate of the Public Health Organization*. Q2.12: How much resources and action are you going to take to screen something, which may also be solved in other ways. I think for example that campaigning information is also a very important aspect, certainly when you talk about costs *– Delegate of the Ministry of Health, Welfare and Sport.*

^1^ Translated from Dutch.

#### Theme 1.2: The test as a screening method

All parents had a positive first impression about using the test as a screening method. Some mentioned it to be a relief in such a way that they might have to travel less to the hospital (quote 1.4). Others noted how difficult it can be to recognize vision loss in children and how their child possibly could have benefitted from earlier detection (quote 1.5). All parents mentioned that they trusted the outcome of the test, even if the outcome would be different from what they would have expected.

#### Theme 1.3: Parties that could distribute the screening method

Most parents named the Dutch Municipal Health Services (MHS; *Gemeentelijke Gezondheidsdienst*) and schools as best options to supervise and distribute the screening program. The MHS was said to be trustworthy, transparent, and well known. The parents were accustomed to receiving information from the MHS. Both parents and patients named schools as a relevant location for supervision and distribution as well since they claimed that teachers are among the first to notice vision loss in children (e.g., difficulties reading the blackboard). Moreover, some children mentioned that school would be the most fun place to take the test, yet most parents also noted that primary schools already are under pressure due to a lack of personnel and might not be able to safekeep medical data (quote 1.6).

#### Theme 1.4: Use case and the uptake of screening by target group

All parents underlined that a part of the target group was expected to refrain from screening, further defined as “non-participators”. They found it important that extra focus and resources would be spent on reaching these non-participators. The reasons for non-participation in their opinion were either practical or motivational. The anticipated practical reasons mentioned were language barriers, low socioeconomic status, or simply being preoccupied. The anticipated motivational reasons were limited awareness on the consequences of myopia, health beliefs that differ from evidence-based medicine, and distrust in safekeeping of medical data (quotes 1.7 and 1.8). The parents noted that increasing awareness might reduce the number of non-participants.

### Perspectives of eye care professionals and policymakers

#### Theme 2.1: The test as a screening method

All stakeholders were supportive of digital screening (quote 2.1). If scientifically proven reliable in children, all eye care professionals mentioned to have trust in the online test being performed by patients and their parents independently of the support of a healthcare professional. They noted that the screening flow was accessible (i.e., steps taken to complete the test), and it especially might have potential in places with a constraint access to care, such as low-income countries.

#### Theme 2.2: Use case and the uptake of screening by target group

All eye care professionals underlined age and the refractive errors as important factors to determine the use case. First, some noted that it is difficult to measure vision in young children (<12 years) due to a shorter attention span, stronger accommodative reflex, and an increased risk of amblyopia (quote 2.2). They suggest that patients ≥12 years were more feasible for screening. Second, patients with higher refractive errors (>2 diopters, D) were expected to have visual complaints and are therefore expected to seek care by themselves, making screening less effective (quote 2.3). The eye care professionals feared that the detection of small refractive errors (e.g., 0.5 D) could result in the unnecessary prescription of glasses, yet they also noted that early detection prevents myopia progression (quotes 2.4 and 2.5). It was additionally suggested that screening might be more effective if solely patients with risk factors were screened, e.g., patients with myopic parents. Lastly, they found it important to identify non-participators, as they argued that this might be a group with higher potential health gains. In addition to the previous arguments given by children and their parents, they expected that unfamiliarity of the Dutch healthcare system could result in non-participation.

#### Theme 2.3: Methodological challenges

All eye care professionals and policymakers underlined the risk for high false-positives (being incorrectly classified as in need of new glasses), potentially resulting in more referrals, unnecessary new glasses, increased costs, and a higher demand for healthcare (quotes 2.6, 2.8, and 2.9). To reduce this effect, the eye care professionals advised that positive tests should be re-evaluated by an optician before being referred (quote 2.7).

#### Theme 2.4: Practical barriers and parties that could distribute the screening method

The identified barriers were the costs of screening and the supervising organization. First, when aligning the screening with the World Health Organization (WHO) and Wilson & Junger screening criteria (9, 10), this program was expected to comply with most criteria, though costs were expected to be more challenging (quote 2.8). The eye care professionals and policymakers anticipated a rise in costs if the false-positive rates were not adequately controlled. The reasons mentioned were increased demand for care and the unnecessary need for patients to purchase glasses (quote 2.9). Second, the eye care professionals and policymakers consistently mentioned two options on how the participants could best be invited for screening: via the MHS and schools. The MHS was preferred, as it is considered trustworthy and safe and has contact information of most children. The schools were advised to be complementary.

#### Theme 2.5: Awareness

Creating awareness was frequently mentioned to be relevant since myopia is significantly impacted by lifestyle choices (quote 2.10). The stakeholders mentioned that the online screening was expected to result in a complementary increased awareness effect (quote 2.11), yet they also underline that a comparable awareness could also be created with a campaign to inform people about the consequences of myopia instead of screening (quote 2.12).

## Discussion

In this qualitative interview study, the barriers and opportunities of using a remote digital screening program for pediatric myopia in The Netherlands, as experienced by myopic patients (aged ≥6 years), their parents, eye care professionals, and policymakers, were analyzed. The test is self-administered and thereby detects refractive errors at home. The results were thematically reported.

Regarding usability, the patients and parents were positive about the instructions and age appropriateness of the test. As improvements, they suggested that the layout of the test could be made more fun for children and they preferred receiving feedback during the test on how well the test instructions were followed (e.g., maintaining correct distance). In line with literature, making a vision test more fun with sounds, cartoons, and other elements is known as “gamification” and can positively impact a child’s motivation, without affecting outcomes (11, 12). Interestingly, none of the interviewees saw it as a barrier that the online test is self-administered (in the direct physical absence of a healthcare professional) as opposed to measuring refractive errors in the clinic. Although this was anticipated to be a barrier, our findings align with literature, concluding that patients are not reluctant to the increased responsibilities due to telemonitoring, which are shifted from healthcare professionals to patients (13).

All interviewees were supportive of the online test as a screening method. The parents noted how difficult it can be to recognize vision loss in children and how their child could have possibly benefitted from earlier detection. Moreover, the patients specifically reported it to be a relief that they might have to travel less to the hospital. High patient satisfaction and acceptance rates are often reported in studies driven by increased accessibility and reduced travel costs and time (14). The eye care professionals and policymakers were also supportive due to the high demand for eye care, highlighting the need to explore alternative options. However, they stress the importance of clearly shown scientific reliability and feasibility. In addition, literature reports that healthcare professionals repeatedly mention other barriers to telemedicine, including the need for trained staff, resistance to change, and concerns about the perception of impersonal care (15).

Regarding the ideal party to distribute the screening method, the MHS was found to be the most reliable partner for the supervision of the screening program by all interviewees since this institution is generally considered trustworthy, transparent, and safe and has contact information of most children. Though all interviewees mentioned schools as a good secondary option, literature reports that telemedicine delivered via schools are much more common, with its popularity steadily increasing (16). This suggests that both options are a valid approach for distribution.

Some eye care professionals noted that it is difficult to measure myopia in young children (<12 years) and therefore this age should be considered an exclusion criterion. Moreover, they expected that screening would be less effective on higher refractive errors, as patients would seek care independently of the screening program due to visual complaints. The experts feared that the detection of small refractive errors (e.g., 0.5 D) could result in the prescription of unnecessary glasses, yet they also argued that early detection combined with, e.g., lifestyle changes could prevent myopic progression compared to no detection or treatment. In addition, all stakeholders had remarks on the fact that they expected that a part of the target population was expected to refrain from screening (i.e., non-participators) even though this is expected to be a group with high potential health gains. Interestingly, an association between digital exclusion and social deprivation with monitoring to smartphone-based self-monitoring has not been found in other studies, suggesting that vision self-monitoring is accessible (17). Nevertheless, the reasons mentioned by the interviewees for non-participation were categorized into those facing structural barriers and those opting out due to personal beliefs and attitudes. This is in line with the study of Vongsachang et al. (18), where the authors examined factors related to decreasing participation in school-based vision programs. Categorizing non-participators helps in finding targeted solutions. Regarding structural barriers, a lower socioeconomic status has long been associated with decreased health, access to healthcare, and attendance to screening programs, even when the financial barriers are minimized (19–23). Though digital health is known to positively influence health equity when implemented properly, recognizing non-participators who are vulnerable remains essential (24, 25). Non-participation due to personal beliefs is often reported to occur due to mistrust in evidence-based medicine, as corroborated in several studies, in which the parents think that glasses weaken the eyes (18, 26, 27). Parental and children’s knowledge of eye health is associated with children undergoing eye examinations and leads to wearing of glasses due to less stigmatization (26, 27). This highlights the importance of awareness in boosting participation. In a qualitative study exploring e-health in adults, it is recommended to focus on building trust, and that access to the healthcare professional is to be retained when indicated or deemed necessary by the patient (28). The strategies suggested are communicating the results clearly and consistently, providing education, and offering logistic support for access to care (26, 27, 29).

Both stakeholder groups expected the costs to be a challenge due to the potential of high false-positive rates not being in balance due to the increased demand for care and patients needing to buy glasses they would not need. This concern underscores the importance of sensitivity and specificity in evaluating digital screening tools. A test with suboptimal specificity may lead to unnecessary referrals and overtreatment, which could undermine trust in the system and result in increased healthcare expenditures. Similarly, insufficient sensitivity may cause missed cases of refractive error, reducing the purpose of early detection. Therefore, while this study focused on stakeholder perspectives, future implementation efforts must carefully consider the diagnostic accuracy of the tool to ensure clinical and economic feasibility.

This occurred previously in Flanders with the *PlusOptix* screening program, leading to uncompensable high societal costs due to high rates of false-positives (30). Therefore, the stakeholders involved in that project suggested a re-evaluation of the positive tests by a local optician before referring to an ophthalmologist to prevent these adverse effects. An alternative solution could be to re-test positive cases by the digital test itself before approaching opticians, as this reduces the false-positives and is considered cost-neutral. It was also proposed to focus screening on patients with risk factors for myopia to increase the effectiveness of the program (e.g., children with myopic parents). *Vice versa*, when screening programs are performed in children, these have the potential to have a major contribution in the light of lifetime costs as is repeatedly reported in interventions for overweight children (31). In turn, apart from the perspective of screening, costs and reimbursements have been most frequently mentioned as barriers for the adoption of telemedicine (15).

Lastly, several stakeholders suggested that raising awareness about myopia through an informative campaign could have a similar effect on screening in terms of prompting parental action and increasing early detection.

Awareness campaigns primarily aim to educate the public about the risks and long-term consequences of myopia, thereby increasing general understanding and prompting a health-seeking behavior. These programs offer several advantages: they are cheap, have the potential to reach a broad audience—including underserved populations—and can empower parents through education. Additionally, they can address common misconceptions (e.g., that glasses weaken the eyes), reduce stigma, and increase the uptake of existing vision services. However, awareness alone lacks objective assessment, making it dependent on parental initiative and the presence of noticeable symptoms. It also introduces risks such as false reassurance, delayed diagnosis, and unequal engagement—especially in asymptomatic children or populations with limited health literacy. In contrast, programs incorporating self-administered tools provide a direct and measurable method for detecting refractive errors that might enable a more timely identification of myopia, regardless of symptom awareness. However, they may come with higher resource demands, risks of false positives, and accessibility challenges in digitally excluded groups. As such, combining awareness efforts with accessible self-screening may create a synergistic effect—raising motivation to engage while simultaneously lowering the barriers to detection and care.

The main idea of early detection to prevent disease in essence is simple and admirable in theory, yet Wilson and Jungner (1968) showed in their landmark study that for a successful implementation, a screening method should uphold all proposed screening criteria. This is in line with suggestions by the interviewed policymakers. Although several adaptations have been made to the criteria since, decades later these criteria stood well against the test of time and are still interpreted as the gold standard of screening assessments. Therefore, barriers identified by the interviewees such as non-participation, false positives, and costs should be assessed carefully before the program is implemented.

### Strengths and considerations

This study benefited from having semi-structured interviews, thus allowing the participants to accentuate, elaborate, and introduce topics. Moreover, we chose to widen the scope by including multiple relevant stakeholder groups, ensuring that barriers and opportunities were validated from different perspectives. All interviewees also had experience in eye healthcare and so had background knowledge to draw upon. With regards to limitations, this study could be prone to both confirmation and selection bias. Confirmation bias should be considered since thematic analysis is subjective and relies on the researchers’ judgment, even though we explicitly asked the interviewees for improvements and pitfalls. Selection bias should be considered as well since patients accepting invitations to participate in research are often more highly educated than those who do not participate. Finally, all patients were treated in a tertiary myopia center, and therefore this selected population might be more positive about eye screening compared to the average population.

## Conclusions

This qualitative stakeholder analysis identified the barriers and opportunities as experienced by patients, eye care professionals, and policymakers of a pediatric vision screening program using a self-administered online eye test. The patients and parents were supportive of screening and suggested improvements. The eye care professionals and policymakers were receptive to screening but also cautious, highlighting concerns about costs and scientific reliability. The policymakers underlined the relevance of fulfilling all screening criteria before implementation, and the eye care professionals recommended to focus on a specific population at risk that benefits most as opposed to screening the whole population. They saw potential in children from an age of 12 years and older without high refractive errors. If the test would be implemented, they recommended to facilitate awareness as a complementary strategy and to re-evaluate positive tests before referring the patients to opticians or eye clinics.

## Data Availability

The raw data supporting the conclusions of this article will be made available by the authors, without undue reservation.
