# Supplementary material for: Stakeholders’ perspectives on a digital myopia screening program in children: a qualitative analysis
Source: Front Ophthalmol (Lausanne). 2025 Jul 8;5:1585320. doi: 10.3389/fopht.2025.1585320 (PMC12279491; doi:10.3389/fopht.2025.1585320)
Supplement: Supplementary file 1 [file Table1.docx]

| Supplementary Table 1. Codebook on identified themes | | | | |
| --- | --- | --- | --- | --- |
| Digital screening | Use case and screening uptake | Methodological design | Practical barriers | Awareness |
| First impression by interviewee  Experience with remote care  Instructions  Support  Added value of digital screening  The future of myopia  Autonomy  Mentality  Independent test without professional  The added value of screening  Current demand on care | Feasibility  Use case  Amblyopia  Myopia screening  Accommodation  Screening uptake  Vulnerable children  Age children  Parental supervision  High and low myopia  Parental supervision | False-positives  Setting  Feasibility  Reliability  Trust in remote care & screening  Groups not reached by screening | Inviting participants  Costs  Commercial interests  Uniformity  Effectiveness  Back-end system & Laws | Preventative solutions  Lifestyle & myopia  Current screening method  Regional vs national screening  Screening criteria  Screening on visual acuity or refraction |
